# The Impact of Lung Function on Sleep Monitoring in Obstructive Sleep Apnea Associated with Obstructive Lung Diseases: Insights from a Clinical Study

**DOI:** 10.3390/jcm13206189

**Published:** 2024-10-17

**Authors:** Antonio Fabozzi, Alessia Steffanina, Ambra Nicolai, Federica Olmati, Matteo Bonini, Paolo Palange

**Affiliations:** Pulmonology Unit, Department of Public Health and Infectious Diseases, Policlinico Umberto I, “Sapienza” University of Rome, 00185 Roma, Italy; alesteffanina@yahoo.it (A.S.); a.nicolai@policlinicoumberto1.it (A.N.); f.olmati@policlinicoumberto1.it (F.O.); matteo.bonini@uniroma1.it (M.B.); paolo.palange@uniroma1.it (P.P.)

**Keywords:** obstructive sleep apnea, sleep apnea syndromes, polysomnography, disorders of excessive somnolence, lung function, chronic obstructive pulmonary disease, asthma

## Abstract

**Background/Objectives:** Obstructive sleep apnea (OSA) and obstructive lung diseases (OLD) are common and interdependent respiratory disorders, where one condition may contribute to the development and worsening of the other (OLDOSA syndrome). The term OLDOSA syndrome includes two different conditions: Overlap syndrome (OVS: OSA + chronic obstructive pulmonary disease, COPD) and Alternative Overlap syndrome (aOVS: OSA + Asthma). Data on the interactions between lung function and respiratory monitoring during sleep in OLDOSA patients are few and controversial. Our study aims to evaluate the impact of lung function impairment on sleep breathing disorders, paying attention to the lack of literature about comparisons between OVS, aOVS, and the impact of small airways disease (SAD) in these patients. **Methods:** In total, 101 patients with a diagnosis of OSA and asthma or COPD underwent pulmonary function tests (PFTs) and nocturnal home sleep cardiorespiratory monitoring (HSCM). Exclusion criteria: Obesity hypoventilation syndrome (OHS) and other non-respiratory sleep disorders. **Results:** Sleep time with oxygen saturation below 90% (T90) was negatively correlated with forced expiratory volume in the first second, % of predicted (%FEV_1_), forced vital capacity, % of predicted (%FVC), forced expiratory flow at 25–75% of the pulmonary volume, % of predicted (%FEF_25–75_), and, after multivariable linear regression analysis, %FEF_25–75_ remained an independent factor for T90 with a negative correlation in mild and moderate OSA. Obstructive apnea index (oAI) and FEV_1_/FVC were negatively correlated in mild and moderate OSA. OVS presented with more severe OSA (higher AHI, oAI, and T90) and SAD (lower FEF_25–75_) compared to aOVS. **Conclusions:** This study highlights a possible interdependence between OLD and OSA; obstruction of the large and small airways at PFTs contributes to the worsening of these patients’ nocturnal hypoxemia and obstructive events of the upper airway during sleep. Furthermore, this study shows that patients with OVS should be carefully monitored, as they present worse data at HSCM and have greater small airways involvement compared to aOVS.

## 1. Introduction

Obstructive sleep apnea (OSA), a condition characterized by repeated episodes of upper airway collapse during sleep, and obstructive lung diseases (OLD) such as asthma or chronic obstructive pulmonary disease (COPD), are common respiratory diseases with high morbidity and mortality [1,2]. OSA and OLD share common risk factors, such as old age, male gender, lower socioeconomic status, cigarette smoking, gastroesophageal reflux disease (GERD), and diabetes [3,4,5,6]. Notably, the interaction between OLD and OSA, commonly referred as OLDOSA syndrome (OLD plus OSA), appears to be reciprocal, with each condition potentially worsening the other [7]. Increased resistance in upper airways (typical of OSA) and lower airways (typical of OLD) seems to contribute to the complex interplay of the OLDOSA syndrome. Indeed, previous studies have demonstrated that, just before an apneic event, lower airway resistance tends to rise, while the cross-sectional area of the upper airway decreases [8,9,10]. Additionally, the reduced elastic recoil, commonly seen in severe asthma or pulmonary emphysema, weakens the support of lower airways, making upper airways more collapsible, especially during sleep.

### 1.1. Overlap Syndrome: Combination of OSA and COPD

Recent data from systematic reviews report a significative prevalence of OSA in COPD patients, the so-called “Overlap syndrome” (range: 56–78%) [11]. In turn, OSA patients present an increased risk of developing COPD, with prevalence rates between 8% and 56% [12]. Key risk factors for OVS in COPD patients are obesity and old age [13], alongside habitual alcohol consumption [14]. Additionally, the use of oral or inhaled steroids in COPD patients increases the risk of OSA due to increased neck fat deposition [15]. The tendency of COPD patients to develop OSA is due to several pathophysiological factors. Reduced diaphragmatic and accessory muscle activity during sleep, combined with the gravitational redistribution of lung mass in the supine position, contribute to increased the upper airway collapsibility in COPD individuals during sleep [16,17]. Additionally, systemic sarcopenia (typical of COPD subjects) can weaken pharyngeal muscles [18]. Nocturnal hypoventilation, seen in up to 43% of COPD patients, becomes more common as airflow limitation worsens [19]. Sleep-related hypoventilation in COPD patients is likely secondary to excessive upper airway resistance and to decreased upper airway dilatory response to hypercapnia, in addition to the previously mentioned decreased diaphragmatic functional activity [16].

### 1.2. Alternative Overlap Syndrome: Combination of OSA and Asthma

The prevalence of asthma in OSA patients is estimated to be around 35% [20], while a recent meta-analysis reported that 50% of asthmatic patients have confirmed OSA [21]. The primary risk factors for OSA in asthmatics include obesity, chronic rhinosinusitis, severe asthma, and GERD [22,23,24]. 

Several pathophysiological mechanisms predispose asthmatic individuals to OSA, leading to the so-called “Alternative Overlap syndrome” (aOVS). First, asthmatic patients exhibit increased parasympathetic tone at night, which triggers bronchial hyperresponsiveness (BHR) and increases airway resistance during sleep [25,26]. Severe asthma is a significant risk factor for the aOVS. Indeed, 60% of patients with severe asthma exhibit chronic neutrophilic inflammation of the airways, which predisposes them to the upper airway collapsibility [27]. In severe asthma, the reduced elastic recoil and the higher reduction in functional residual capacity (FRC) during sleep disrupt the mechanical coupling between airways and parenchyma, leading to the inhibition of the stiffening effect of “tracheal tug”, which results in increased nocturnal upper airways collapsibility [28,29,30].

### 1.3. Lung Function and OSA in Obstructive Lung Diseases

Evidence from the literature suggests that individuals with reduced lung function are at a higher risk for OSA. However, this increased risk is often attributed to confounding factors such as age and obesity, rather than a direct association between reduced lung function and OSA [31]. A recent study about lung function and OSA risk demonstrated that lower levels of forced vital capacity (FVC), forced expiratory volume in the first second (FEV_1_), and FEV_1_/FVC are more common in the OSA high-risk patients. However, after adjusting for BMI, these pulmonary function test (PFT) parameters did show an independent association with OSA risk [32]. Conversely, another study found that OSA patients exhibit a more rapid decline for FEV_1_ and FVC compared to the general population, with asthmatic patients being particularly affected [33]. In addition, another study reported an accelerated annual decline in %FEV_1_ in asthmatic patients with OSA compared to those without OSA. In patients with severe OSA, continuous positive airways pressure (CPAP) therapy decelerated the annual decline in %FEV_1_ [34]. To this date, there is controversy regarding the correlation between lung function parameters (such as FEV_1_) and polysomnographic parameters, such as apnea–hypopnea index (AHI) in patients with OVS and aOVS. Indeed, some studies show that increased end-expiratory lung volume (EELV) in COPD patients may offer protection against upper airway closure during sleep [35,36]. Other studies showed that a lower FEV_1_ may play some protective role for the OSA severity in patients with OVS [37]. In contrast, in a Japanese outpatient cohort of individuals with aOVS, %FEV_1_ and %FVC were negatively correlated with AHI [38].

### 1.4. Aim of the Study

The rationale for this study stems from the existing controversies in the literature regarding obstructive lung diseases and obstructive sleep apnea, as well as the lack of data on the impact of lung function on sleep breathing disorders’ indices between patients with OVS and aOVS. Thus, this study aims to identify whether large and small airways obstruction at PFTs negatively influences home sleep cardiorespiratory monitoring (HSCM) indexes in OLDOSA patients, and if this relationship changes according to the severity of OSA. Additionally, we seek to investigate differences in lung volumes and nocturnal sleep breathing parameters between OVS and aOVS in order to better understand if this relationship may improve the management of OLDOSA patients.

## 2. Materials and Methods

We conducted a retrospective, single-center, real-life observational study performed at the respiratory sleep disorder center of Pneumology, Policlinico Umberto I (Rome, Italy). The sample size was obtained by retrospectively analyzing the medical files of all 309 patients referred for first medical visit to our center from January 2020 to January 2024, of whom 101 patients met the inclusion and exclusion criteria. Data from the initial visit, including PFTs and HSCM, were collected. HSCM was performed following the guidelines set by the American Academy of Sleep Medicine (AASM) [39] using SOMNO touch RESP device (SOMNOmedics Italia, Ora, Bolzano, Italy) and DOMINO light software (V1.5.0.12) (SOMNOmedics Italia, Ora, Bolzano, Italy) [40]. HSCM consisted of eight integrated channels: nasal flow, snoring sensor, thoracic effort, abdomen effort, oxygen saturation SpO_2_, pulse rate, plethysmogram, and body activity. PFTs were performed by expert staff using a spirometer (Quark PFT, Cosmed, Pavona, Italy), following the recommendations of the American Thoracic Society and European Respiratory Society [41]. The bronchodilator reversibility was performed 15 min after salbutamol 400 μg. Inclusion criteria included age ≥ 18 years and a diagnosis of OSAS (AHI > 5 events per hour with disability-related symptomatology or AHI > 15 events per hour) and a previous diagnosis of asthma or COPD using the respective international guidelines [3,42]. A post-bronchodilator FEV_1_/FVC < 0.7 was diagnosed as COPD [3], and a post-bronchodilator FEV_1_/FVC < 0.75 with an increase in FEV_1_ of 12% and >200 mL was diagnosed as asthma [42]. COPD and asthmatic patients were under treatment, as recommended by the respective international guidelines [3,42]. Exclusion criteria included a diagnosis of other non-respiratory sleep disorders or a diagnosis of obesity hypoventilation syndrome (OHS). The following parameters were assessed: age, body mass index (BMI), smoking history, Epworth sleepiness scale (ESS), AHI, central apnea index (cAI), obstructive apnea index (oAI), mixed apnea index (mAI), HI (hypopnea index), oxygen-desaturation index (ODI), proportion of cumulative sleep time with oxygen saturation below 90% (T90), FEV_1_/FVC (Tiffeneau index), FEV_1_ and FVC (in % of predicted and in liters), and forced expiratory flow at 25–75% of the pulmonary volume (%FEF_25–75_, in % of predicted and in liters per second). According to the manual for the scoring of sleep and associated events by AASM, apnea was defined as a reduction in the peak signal excursion by ≥90% from the pre-event baseline for ≥10 s using a nasal flowmeter sensor [39]. Hypopnea was defined as a reduction in the peak signal excursion by ≥30% from the pre-event baseline for ≥10 s, accompanied by ≥3% arterial oxygen desaturation [39].

### Statistical Analysis

Results are reported as mean with standard deviation (SD). To compare the means of independent variables, we employed one-way analysis of variance (ANOVA) and Student’s *t*-test, while Pearson’s correlation coefficient was applied to evaluate the linear correlation between variables. The chi-square test was utilized to examine the prevalence of variables between the two groups. Statistical significance was established at *p* < 0.05. All data analyses were conducted using Jamovi software (V2.3.28.0).

## 3. Results

In total, 101 patients (30 women and 71 men) were evaluated (Table 1). The cohort showed a high prevalence of Class I Obesity (mean BMI: 32 ± 7 kg/m^2^), and a majority were males (70%). Mean ESS score was 13 ± 5, which is above the positivity threshold. The mean FEV_1_ was 76 ± 21% of predicted, indicating a mild obstructive ventilatory deficit. In addition, the mean FEF_25–75_ (49 ± 19% of predicted) suggested a moderate ventilatory impairment of small airways. Both ESS and BMI were significantly and positively correlated with AHI, ODI, and T90. In particular, ESS was positively correlated with AHI (r = 0.35, *p* < 0.001), ODI (r = 0.38, *p* < 0.001), and T90 (r = 0.28, *p* = 0.004); furthermore, BMI was positively correlated with AHI (r = 0.48, *p*< 0.001), ODI (r = 0.5, *p* < 0.001), and T90 (r = 0.36, *p* < 0.001). A significantly negative correlation was found between T90 and PFTs parameters, in particular %FEV_1_ (r = −0.2 and *p* = 0.04), FVC, and %FVC (r = −0.2 and *p* = 0.04), and FEF_25–75_ and %FEF_25–75_ (r = −0.2 and *p* = 0.04).

**Figure 1:**
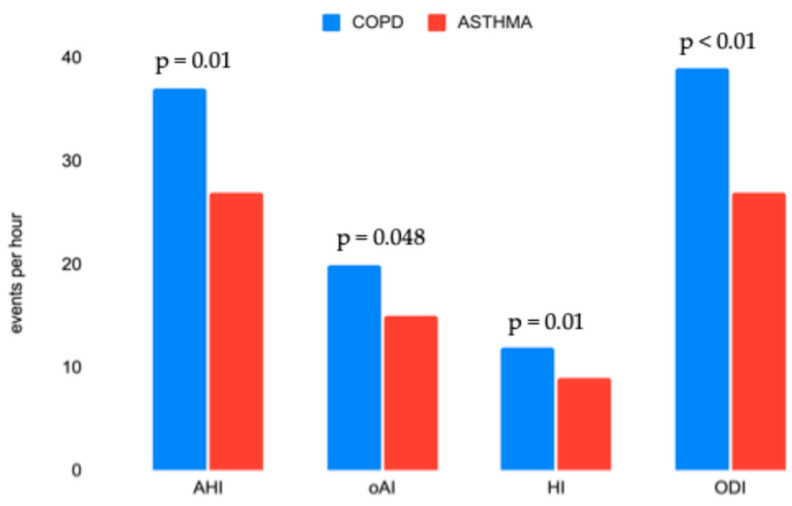
HSCM parameters resulted significantly different between COPD (OVS) and asthma (aOVS). All four HSCM parameters are statistically significant. AHI, apnea–hypopnea index; oAI, obstructive apnea index; HI, hypopnea index; ODI, oxygen desaturation index.

In addition, we conducted a sub-analysis dividing patients into the two groups previously described: OVS and aOVS (Table 2). In the OVS group, the prevalence of men was much higher compared to aOVS (t = 4.4, *p* = 0.035). Furthermore, OVS patients showed significantly worse HSCM parameters in respect to the aOVS group (Figure 1), specifically, AHI (t = 6, *p* = 0.01), oAI (t = −1.7. *p* = 0.048), ODI (t = −2.6, *p* = 0.004), and T90 (t = −4.5, *p* < 0.001). FEF_25–75_ was the only PFTs parameter that showed a statistically significant difference between the two groups. OVS patients had a mean FEF_25–75_ of 1.4 ± 0.7 L/s, while aOVS patients had a mean FEF_25–75_ of 1.6 ± 0.7 L/s (t = 1.7, *p* = 0.04).

**Figure 2:**
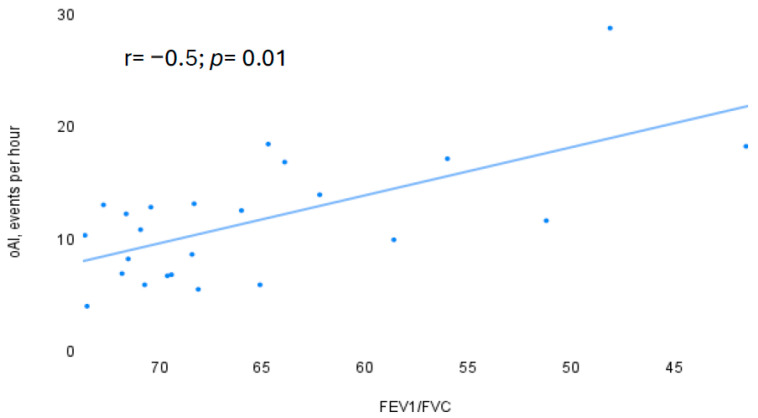
Significant linearly negative correlation between oAI and FEV_1_/FVC in mild and moderate OSA. Figure 2 oAI, obstructive apnea index; FEV_1_, forced expiratory volume in the first second; FVC, forced vital capacity.

Stratifying our patients according to AHI, we divided them into three groups (Table 3): mild OSA (AHI < 15 events per hour), moderate OSA (AHI between 15 and 30 events per hour), and severe OSA (AHI > 30 events per hour). Among these groups, no significant differences were observed, except to BMI and ESS, which were significantly higher in the severe OSA group (BMI: F = 8.6, *p* < 0.001; ESS: F = 5.8, *p* = 0.004). One notable finding from this sub-analysis was that a statistically significant negative correlation was found between oAI and FEV_1_/FVC only in mild and moderate OSA (r = −0.5, *p* = 0.01), but this correlation disappeared in the severe OSA group (Figure 2).

Finally, a multivariable linear regression analysis for HSCM parameters in severe OSA did not show statistically significant results. However, in mild and moderate OSA groups, several relevant findings were observed (Table 4). BMI was independently associated with AHI, ODI, and T90, all showing a significant positive correlation; FEV_1_/FVC was independently and negatively associated with oAI, and %FEF_25–75_ was independently associated with T90, showing a significant negative correlation.

## 4. Discussion

The findings from our study suggest a complex but significant correlation between PFTs and HSCM parameters in OLDOSA patients, suggesting a synergy between OLD and OSA.

### 4.1. Impact of BMI and ESS on Sleep Parameters

The positive linear correlation between BMI and key HSCM parameters (AHI, ODI, and T90) confirms that obesity is a critical contributor to OSA severity, even in patients with underlying obstructive lung diseases. These results are consistent with the previous literature, where BMI has been identified as a significant predictor of OSA in patients with COPD or asthma [37,38,43]. Similarly, the mean ESS of 13 ± 5 points and its positive correlation with AHI, ODI, and T90 suggest that excessive daytime sleepiness (EDS) is common in our cohort, in accordance with previous studies where ESS proved to be an independent predictor of OSA in asthma or COPD [34,37,38,44].

### 4.2. Lung Function and Nocturnal Hypoxemia

A particularly notable finding was the significant negative correlation between T90 and %FEV_1_, %FVC, and %FEF_25–75_. In fact, this result may suggest a possible synergistic effect between upper and lower airways obstruction, leading to more pronounced hypoxemia in OLDOSA patients when they have a significant decline in %FEV_1_, %FVC, or %FEF_25–75._ However, a multivariable linear regression analysis revealed that %FEF_25–75_ was the only independent negative factor for T90, suggesting that small airways impairment may deteriorate nocturnal hypoxemia in OLDOSA subjects.

### 4.3. Influence of Lower Airways Obstruction on Sleep Apnea

The significant negative correlation between FEV_1_/FVC and the obstructive apneas index (oAI) in patients with mild and moderate OSA suggests that lower airways obstruction may contribute to more obstructive apneic events during sleep in OLDOSA patients. This correlation was supported by multivariable regression analysis, where FEV_1_/FVC was a significant independent negative factor for oAI in mild and moderate OSA, both in COPD and asthmatic patients. However, this correlation loses significance in patients with severe OSA, as the high AHI diminishes the impact of lung function on the development of OSA. These findings partially align with the previous literature, where %FEV_1_ was negatively correlated with T90 in Overlap syndrome. However, to our knowledge, no previous study has examined the correlation between HSCM parameters and small airways PFTs parameters, such as FEF_25–75_ [37]. As demonstrated by Spina G. et al., severe airflow limitation at PFTs in COPD patients was associated with more fragmented sleep and reduced sleep efficiency, as measured by nocturnal actigraphy [45]. Previous studies have shown a negative correlation between OSA severity (measured by AHI) and PFTs parameters (FEV_1_, FVC) in aOVS [38], while OVS patients demonstrated a positive correlation between AHI and FEV_1_ [37]. In our study, AHI was not correlated with any lung function parameters. This discordant relationship between AHI and FEV_1_ suggests that FEV_1_ might be replaced by other parameters that measure alveolar hyperinflation, such as EELV or inspiratory capacity/total lung capacity (IC/TLC), while AHI could be replaced by oAI, as we showed through the association between oAI and FEV_1_/FVC [46,47]. FEV_1, however,_ has shown a significant role in previous studies for evaluating the decline of lung function over time in aOVS patients, decreasing in an AHI-dependent manner compared to asthmatic patients without OSA [34].

### 4.4. The Impact of Age on OLDOSA Syndrome

The mean age of our cohort was 71 years, with no differences between OVS and aOVS and between mild, moderate, and severe OSA. This elevated mean age indicates that our findings are based on an elderly population, which may make the application of our results to younger populations more challenging. Aging is a key risk factor for OSA, as the upper airway naturally narrows and fat deposition physiologically increases with aging, resulting in higher pharyngeal resistance [48]. In addition, the elderly are predisposed to the phenomenon of overnight rostral fluid shift, which facilitates upper airway closure in the supine position [49]. In our study, however, multivariate analysis did not show that age was a predictive factor for HSCM parameters.

### 4.5. Overlap Syndrome and Alternative Overlap Syndrome: The Comparison

To the best of our knowledge, this is the first study comparing lung function and HSCM parameters between patients with OVS (OSA + COPD) and aOVS (OSA + asthma). We found that OVS patients had higher values in key HSCM parameters (AHI, oAI, HI, ODI, and T90) compared to aOVS patients, suggesting that OSA has a more severe impact on COPD patients compared to asthmatics. The pathophysiological mechanisms that could underlie the higher severity of OSA in COPD patients may include greater alveolar hyperinflation with an increase in EELV, increased small airways impairment (as demonstrated by us with FEF_25–75_), reduced ventilatory response to hypoxia and hypercapnia during sleep, and greater weakening of respiratory muscles, including both diaphragm and oropharyngeal muscles [19,47,50,51,52]. Furthermore, the more severe impairment of small airways in OVS compared to aOVS is consistent with the previous literature, which reported a more severe small airways disease (SAD) in COPD compared to asthma, using FEF_25–75_ as the primary indicator [53].

### 4.6. Clinical Implications

First, the two underlying obstructive lung diseases should be early distinguished, as COPD patients present more severe OSA and may need more intensive treatments, such as CPAP or nocturnal non-invasive ventilation (NIV), from the beginning. Furthermore, it is critical to perform early screening through appropriate questionnaires for OLDOSA syndrome in these subjects. This type of patients should also be monitored more closely during follow-up, both with HSCM and PFTs, as a decline in %FEV_1_ could indicate poor OSA control, while an increase in AHI during follow-up could correspond to poor control of the obstructive lung disease.

### 4.7. Therapeutic Implications

Further research is needed to investigate whether effective management of OLD with bronchodilator therapies, anti-inflammatory therapies (such as anti-leukotriene receptors for asthma), or biological therapies could slow the progression of nocturnal hypoxemia and obstructive sleep apneas in OLDOSA patients. One potential therapeutic strategy might be the use of evening long-acting bronchodilation. Indeed, in a clinical trial by Domnik NJ et al., COPD patients who underwent evening long-acting bronchodilation showed improved overnight dynamic respiratory mechanics by reducing total airways resistance and lung hyperinflation [54]. A possible future direction might be to evaluate the impact of evening long-acting bronchodilation on HSCM parameters in OLDOSA syndrome. Additionally, the impact of early CPAP or NIV on lung function of OLDOSA patients should be explored, as well as whether differences in PFTs outcomes exist between these treatments. Furthermore, this study highlights the need for personalized treatment approaches for these patients, as well as the development of specific guidelines for the management of OLDOSA patients, based on evidence-based medicine (EBM).

### 4.8. Study Strengths and Limitations

The strength of our study lies in the investigation and comparation of HSCM and PFTs in OLDOSA patients, differentiating them into COPD or asthma subjects. This allowed us to study all possible interactions and the impact of lung function on respiratory events during sleep. Notably, we evaluated the impact of small airways impairment on nocturnal hypoxemia in OLDOSA syndrome, a novel finding in the literature. 

However, possible limits of the present study include its retrospective and observational nature. The small sample size and the single-center nature of the study could affect the generalizability of the results and their application to daily clinical practice. Additionally, since the data collected in our study pertain to baseline measurements (the time of diagnosis of the two diseases), the lack of follow-up data represents a limitation, preventing us from assessing the long-term effect of therapies such as CPAP/NIV for OSA and bronchodilator or biologic therapies for obstructive lung disease. Furthermore, this study did not include anatomical measurements (neck circumference, diameter of the oropharynx, and neck fat on magnetic resonance imaging). Finally, the absence of parameters such as TLC, residual volume (RV), or IC did not allow us to evaluate the effect of lung hyperinflation on respiratory events during sleep in OLDOSA patients.

## 5. Conclusions

In conclusion, our findings in OLDOSA patients suggest a complex relationship between lung function and sleep monitoring. This was demonstrated by the correlation between FEV_1_, FVC, and FEF_25–75_ with T90, which is considered the best index of hypoxemia during sleep. In addition, we hypothesize that lower airway obstruction (typical of OLD) may predispose patients to more obstructive apnea events during sleep, as indicated by the negative correlation between FEV_1_/FVC and oAI and from linear multivariable regression analysis. By subdividing the patients into OVS and aOVS, we observed that COPD patients exhibited worse HSCM parameters during sleep compared to asthmatic patients, which highlights the need to focus on these specific patients. Finally, we also explored the association between small airways and nocturnal hypoxemia, showing that small airways impairment aggravated nocturnal hypoxemia in OLDOSA subjects. However, both conditions may independently contribute to nocturnal hypoxemia, so we cannot definitively conclude that the correlation between OLD and OSA is the direct cause of worsening nocturnal hypoxemia. Further prospective studies are needed to clarify these aspects. This study emphasizes the need for careful monitoring of OLD patients with mild to moderate OSA, particularly those with impaired lung function, to prevent the progression of both diseases. Early diagnosis and intervention are crucial for improving overall health and quality of life. Further prospective studies are needed to evaluate the role of pharmacological therapy, ventilatory support, and weight-control interventions in these patients.

## Figures and Tables

**Table 1:** Parameters and patient’s demographic and functional characteristics.

	Number of Patients: 101
Men/Women, *n* (%)	71 (70)/30 (30)
Age, years	70 ± 11
Smoking habit, *n* (%)	No smokers: 28 (28)
	Smokers: 17 (17)
	Former smokers: 56 (55)
Asthma, *n* (%)	35 (34)
COPD, *n* (%)	66 (65)
BMI, kg/m^2^	32 ± 7
ESS	13 ± 5
AHI, events per hour	34 ± 19
oAI, events per hour	18 ± 14
T90, %	19 ± 20%
ODI, events per hour	35 ± 21
FEV_1_/FVC	65 ± 6
FEV_1_, % of predicted	76 ± 21
FVC, % of predicted	90 ± 21
FEF_25–75_, % of predicted	49 ± 19

Table 1 values are means ± standard deviation. COPD, chronic obstructive pulmonary disease; BMI, body mass index; ESS, Epworth sleepiness scale; AHI, apnea–hypopnea index; oAI, obstructive apnea index; T90, sleep time with oxygen saturation below 90%; ODI, oxygen desaturation index; FEV_1_, forced expiratory volume in the first second; FVC, forced vital capacity; FEF_25–75,_ forced expiratory flow at 25–75% of the pulmonary volume.

**Table 2:** Parameters and patients’ characteristics in Overlap syndrome vs. Alternative Overlap syndrome.

	Overlap Syndrome (*n* = 66)	Alternative Overlap Syndrome (*n* = 35)	*p* Value
Men/Women, *n* (%)	51 (77)/15 (23)	20 (57)/15 (43)	0.035
Age, years	71 ± 9	68 ± 14	NS
	No smokers: 6 (9)	No smokers: 23 (66)	
Smoking habit, *n* (%)	Smokers: 21 (32)	Smokers: 8 (23)	<0.001
	Former smokers: 39 (59)	Former smokers: 4 (11)	
BMI, kg/m^2^	32 ± 7	31 ± 7	NS
ESS	13 ± 5	12 ± 5	NS
AHI, events per hour	37 ± 20	27 ± 16	0.01
oAI, events per hour	20 ± 15	15 ± 11	0.048
T90, %	16 ± 21%	8 ± 10%	<0.001
ODI, events per hour	39 ± 22	27 ± 18	0.004
FEV_1_/FVC	64 ± 6	65 ± 6	NS
FEV_1_, % of predicted	77 ± 23	76 ± 19	NS
FVC, % of predicted	90 ± 22	90 ± 19	NS
FEF_25–75_, % of predicted	48 ± 19	50 ± 17	NS
FEF_25–75_, liters per second	1.4 ± 0.7	1.6 ± 0.7	0.04

Table 2 values are means ± standard deviation. COPD, chronic obstructive pulmonary disease; BMI, body mass index; ESS, Epworth sleepiness scale; AHI, apnea–hypopnea index; oAI, obstructive apnea index; T90, sleep time with oxygen saturation below 90%; ODI, oxygen desaturation index; FEV_1_, forced expiratory volume in the first second; FVC, forced vital capacity; FEF_25–75_, forced expiratory flow at 25–75% of the pulmonary volume; NS, not significant.

**Table 3:** Parameters and patients’ characteristics in mild, moderate, and severe OSA.

	Mild OSA (*n* = 19)	Moderate OSA (*n* = 26)	Severe OSA (*n* = 56)	*p* Value
Men/Women, *n* (%)	11 (58)/8 (42)	19 (73)/7 (27)	41 (74)/15 (26)	NS
Age, years	67 ± 16	69 ± 11	71 ± 9	NS
Smoking habit, *n* (%)	No smokers: 6 (32)	No smokers: 9 (35)	No smokers: 14 (24)	NS
	Smokers: 5 (26)	Smokers: 9 (35)	Smokers: 17 (30)	
	Former smokers: 8 (42)	Former smokers: 8 (30)	Former smokers: 25 (46)	
COPD, *n* (%)	9 (53)	16 (62)	41 (73)	NS
Asthma, *n* (%)	8 (47)	10 (38)	15 (27)	NS
BMI, kg/m^2^	28 ± 4	29 ± 6	34 ± 8	<0.001
ESS	10 ± 6	11 ± 4	14 ± 5	0.004
FEV_1_/FVC	65 ± 6	65 ± 5	66 ± 5	NS
FEV_1_, % of predicted	77 ± 19	73 ± 25	78 ± 20	NS
FVC, % of predicted	94 ± 17	87 ± 24	90 ± 21	NS
FEF_25–75_, % of predicted	50 ± 21	47 ± 23	49 ± 16	NS

Table 3 values are means ± standard deviation. COPD, chronic obstructive pulmonary disease; BMI, body mass index; ESS, Epworth sleepiness scale; FEV_1_, forced expiratory volume in the first second; FVC, forced vital capacity; FEF_25–75_, forced expiratory flow at 25–75% of the pulmonary volume; NS, not significant.

**Table 4:** Multivariable linear regression analysis for AHI, oAI, ODI and T90 in mild and moderate OSA (*n* = 45).

Dependent Variable	Independent Variable	β Coefficient	OR (95% CI)	*p* Value
AHI	BMI	0.597	1.819 (1.061–3.092)	0.03
oAI	FEV_1_/FVC	−0.511	1.667 (1.029–2.699)	0.035
ODI	BMI	1.14	3.13 (1.668–5.868)	0.003
T90	BMI	1.83	6.238 (2.832–13.783)	0.001
T90	%FEF_25–75_	−0.46	0.630 (0.420–0.945)	0.04

Table 4 only statistically significant results have been included in this table. OR odds ratio; BMI, body mass index; AHI, apnea–hypopnea index; oAI, obstructive apnea index; ODI, oxygen desaturation index; T90, sleep time with oxygen saturation below 90%; FEF_25–75_, forced expiratory flow at 25–75% of the pulmonary volume (% of predicted); NS, not significant.

## Data Availability

The dataset used for our analysis are available upon demand to the corresponding author of this study.
